# Magnetic Compression Anastomosis in Long-Gap Esophageal Atresia Gross Type A: A Case Report

**DOI:** 10.1055/s-0038-1649489

**Published:** 2018-05-23

**Authors:** Mark Bremholm Bremholm Ellebaek, Niels Qvist, Lars Rasmussen

**Affiliations:** 1 Department of Surgery, Odense University Hospital, Odense, Denmark

**Keywords:** esophageal atresia, long gap, magnamosis

## Abstract

Esophageal atresia (EA) Gross type A (long-gap without tracheoesophageal fistula) is a rare and a surgical challenging form of EA that constitutes ∼6% of the children born with EA. We present the seventh reported case with successful esophagoesophagostomy obtained by magnetic compression of a long-gap EA type A without thoracotomy.

## Introduction

Esophageal atresia (EA) Gross type A (long gap without tracheoesophageal fistula) is a rare and surgical challenging variation in EA that constitutes ∼7% 1 of the children born with EA. A primary esophagoesophagostomy will almost always be impossible, and several surgical techniques to establish the continuity of the gut have been developed with small or large intestinal interposition, gastric tube or pull-up, and active elongation of the pouches as the most common. 2 All procedures carry an inherent high risk of postoperative complications and long-term functional problems. The incidence of subclinical musculoskeletal deformities after EA repair with muscle-sparing thoracotomy has recently been reported as high as 25%. 3 We present a case with successful delayed esophagoesophagostomy obtained by magnetic compression of a long-gap EA type A without thoracotomy.

## Case Report

The boy was delivered in gestational week 32 + 5 by emergency cesarean section due to leaking amniotic fluid. Birth weight was 1920 g, and Apgar score at 1, 5, and 10 minutes was 8, 5, and 10, respectively. Prenatal ultrasound screening had given the suspicion of EA due to absence of a gastric bubble and polyhydramnios. The diagnosis was confirmed at birth by failure to place a gastric tube and plain abdominal X-ray showed no visible gas in the stomach or intestine compatible with type A EA based on Gross classification. 4 No other congenital malformation was found at general physical examination, echocardiography, or ultrasound of the cerebrum.

**Fig. 1:**
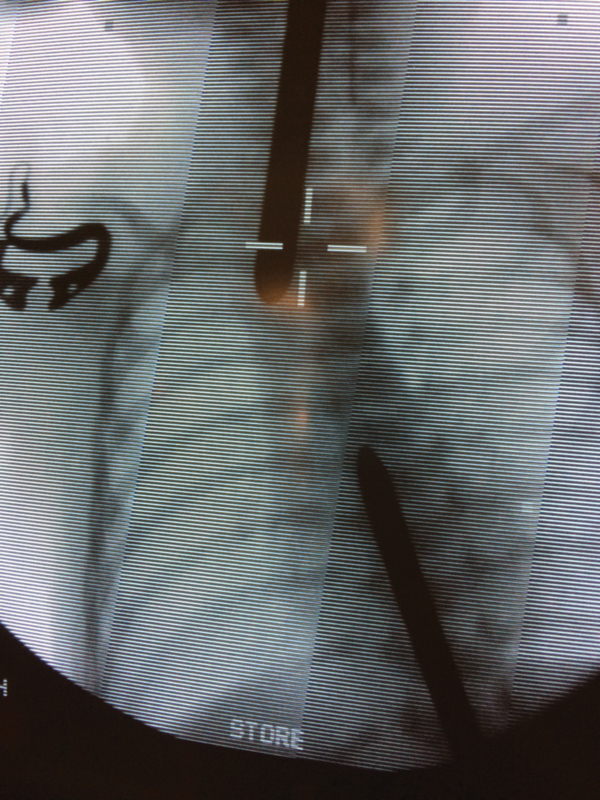
Anteroposterior X-ray demonstrating measurement of the esophageal gap on day 1 after birth.

One-day old a Ch 14 gastrostomy tube (Flocare, Nutricia, Denmark) was placed by laparotomy and the distance between the upper and lower pouch was measured by the Hegar method and was found to be equivalent to the height of 3.5 thoracic vertebrae ( Fig. 1 ). Postoperative permanent suction of the upper pouch was instituted and spontaneous growth of the lower and upper pouch was awaited. Full enteral nutrition via the gastrostomy was launched as boluses. The parents gently pushed on the esophageal tube daily to stimulate elongation and growth of the upper esophageal pouch.

**Fig. 2:**
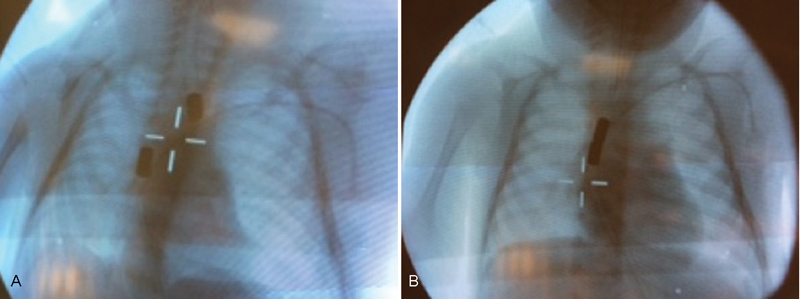
( **A** ) A 5 mm cylindrical magnet (12,000 G) was placed in the upper pouch and lower pouch under fluoroscopic guidance at the age of 62 days. ( **B** ) Immediate contact and alignment between the magnets was observed.

At the age of 54 days, bodyweight was 3,100 g and the distance between the upper and lower pouch was measured to be less than 5 mm. After several information sessions with the parents, it was decided in agreement to try to approximate the two pouches by magnet force as previously reported. The parents were informed about the possibilities for a surgical approximation with the pros and cons including the risk of stenosis in relation to the magnetic method. 5 6 Two identical cylindrical magnets with a diameter of 5 mm and a strength of 12,000 G (Hindsbo Magneter ApS, Roskilde, Denmark) were mounted and secured in the tip of two separate gastric tubes CH 18 (Biofarma Logistik A/S, Glostrup, Denmark). The magnets were specially designed for the purpose with a power similar to other reports. The magnets were sterilized prior to use. Ethical approval was not necessary according to Danish law. At the age of 62 days, each magnet was placed intraluminal at the tip of each pouch under fluoroscopic guidance ( Fig. 2 ), the lower tube via the gastrostomy tube and the upper peroral. Contact and alignment between the magnets was achieved after 15 minutes. Enteral nutrition was continued via the lower pouch tube where a side hole was created corresponding to the part located in the stomach. A temporary increase in leucocyte count in peripheral blood to a maximum of 19.8 10 ^9^ /L (reference values 6.0–13.3 × 10 ^9^ /L) and C-reactive protein to 106 mg/L (reference value < 10 mg/L) was observed and normalized within 3 days. No antibiotics were administered. On postoperative day 5, the tubes including the magnets were removed and replaced with a nasogastric tube without any problems. Enteral nutrition was changed to the nasogastric tube and the gastrostomy tube was removed. Peroral nutrition was tolerated on day 10. On day 16, the boy underwent esophagoscopy due to dysphagia. A stenosis was found, which was dilated (5 mm balloon). Early and frequent dilations at any symptoms on stenosis were decided to prevent fibrous stenosis that might become refractory to dilatation with the need of stenting or surgery. Thus, during the first 3 months, 12 endoscopic dilations where performed. During the next 9 months, additional five endoscopic dilations were performed. The child is now 15 months old and we have not seen him for dilation for 3 months. Otherwise normal psychomotor development and normal height and weight gain according to standard curve for Danish children were observed.

## Discussion

This is the seventh report case of using magnamosis in the repair of a patient with type A EA without any other surgical interventions than the placement of a gastrostomy tube. In the previous cases, the magnets and tube system from Cook Medical have been used. 7 The equipment used in the current case is noncommercial. The principles of the two systems are almost equal with some minor difference. Our system did not include the ability for suction of saliva from the upper pouch and the shape of the magnets used in the previous cases was bullet-shaped opposite cylindrical shaped magnets in the present case. The strength of the magnets was equal around 12,000 G.

In the present case, an elongation of the pouches occurred within 2 months to obtain a distance of 5 mm, which we considered sufficient for applying the method. To promote growth, it is our practice that parents are instructed to gently push the tube in the upper pouch daily, which in theory may facilitate growth. There is no evidence of how short the distance between the pouches must be, but magnamosis has been suggested in gaps up to 3 cm. 6 The presented equipment in its present form is not considered suitable for such large gaps due to the lack of control over the magnetic contraction and thereby the risk of perforation in one or both pouch.

Magnetic contact occurred almost immediately and the anastomosis was achieved within 5th postoperative day in the present cases, which is comparable to the previously reported cases where the average was 4.2 days (range 3–6 days). 6

One of the advantages for nonsurgical magnamosis is the avoidance of the extensive mobilization and dissection of the pouches with the risk of tracheal injury, devascularization, denervation of the esophagus, and the long-term consequences of thoracotomy. 5 A disadvantage with the method might be high rate of early anastomotic stenosis, as seen in the present and previously published cases, which requires early and frequent dilation. Some strictures are persistent and require stenting or surgical reconstruction. 7 Another disadvantage is the waiting period of 2 to 3 months for the natural growth of the esophageal pouches 8 that may require long hospital stay and the constant risk of aspiration pneumonia. These factors must be balanced against the disadvantages of esophageal replacement that seems to have more long-term complications compared with delayed primary anastomosis when considering the method. 9 10 In the presented case, a thoracoscopic repair with its advantages in terms of avoiding musculoskeletal morbidity could have been another solution due to the short distance of 5 mm after the waiting period. This method might have reduced the number of dilatations.

Almost only positive results have been reported regarding the use of magnamosis and one could suspect some publication bias and thus lack of reporting postoperative complications to the method.

For the magnamosis principle, further design refinements are necessary especially to reduce the rate of postanastomotic stenosis. The shape and size compared with the esophageal diameter and strength of magnets may be important. The method could be a promising modality in selected cases with a long-gap EA and were a significant spontaneous growth and elongation of the pouches occurs within a few weeks postnatal. Further investigation and refinement of the method is required before the method can be recommended in general.
